# Emotional Intelligence and Mindfulness: Relation and Enhancement in the Classroom With Adolescents

**DOI:** 10.3389/fpsyg.2018.02162

**Published:** 2018-11-09

**Authors:** César Rodríguez-Ledo, Santos Orejudo, Maria Jesús Cardoso, Álvaro Balaguer, Javier Zarza-Alzugaray

**Affiliations:** ^1^Department of Educational Sciences, University of Zaragoza, Zaragoza, Spain; ^2^Department of Psychology and Sociology, University of Zaragoza, Zaragoza, Spain; ^3^Department of Learning and Curriculum, University of Navarre, Pamplona, Spain; ^4^Department of Musical and Body Expression, Complutense University of Madrid, Madrid, Spain

**Keywords:** mindfulness, emotional intelligence, program implementation, enhancement, adolescents

## Abstract

Emotional intelligence (EI) and mindfulness are two constructs that have been separately studied, and the relation between them still remains unclear. Research in this area has not attempted to go further into how enhancing EI and mindfulness together can achieve better improvements in this ability to attend mindfully. To bridge this knowledge gap, our research goal was to study the relationship between EI and the mindfulness competence in our study sample and to assess the impact of implementing EI and a mindfulness competence developmental program (SEA) about participants’ mindfulness competence. The sample consisted of 156 students aged 11–14 years old from a Spanish public high school. One hundred and eight participants were randomly assigned to the experimental condition, and the remaining 48 were to the control condition. The instruments used to evaluate EI were the CDE-SEC, EQi-Youth Version and the General Empathy Scale. Mindfulness on the School Scope Scale was used to assess mindfulness competences. Social adaptation was evaluated by using the social abilities and adjustment questionnaire BAS3. All the instruments where answered by the participants and have been adapted to a sample of youths with such age specifications. The results showed that EI and mindfulness were related to many of the variables measured by the instruments. Showing a good mindfulness competence was particularly related to having a good general level of the EI trait, and to many of the assessed social and emotional variables. The data indicated a significant relation between the mindfulness competence and having better general empathy skills or being better socially adjusted to the school context. The data also indicated a significant effect on participants’ interior and kinesthetic mindfulness competence after implementing the SEA Program. These findings corroborate the relationship between EI and mindfulness, and the possibility of enhancing mindfulness by applying a direct intervention program in the classroom.

## Introduction

Interest in how emotional intelligence (EI) can influence academic, professional and social success in a person’s development has significantly increased in recent years (Collaborative for Academic Social and Emotional Learning [CASEL], 2015). The social and emotional learning (SEL) concept has been defined as educational innovation that is justified in social needs whose purpose is to develop emotional competences that contribute to better personal and social well-being (Bisquerra, 2003). This interest has led many researchers to study the theoretical basis of the construct and its components. Another focal point for researchers in the field has been to study the best way to enhance EI through training. In this regard, assessing the advantages of enhancing EI components in the classroom has become a mayor study field. Moreover, the relation between EI and other possibly related constructs has been found to be intimately linked to emotional development. One of these constructs is mindfulness, whose relationship with EI has received little attention. As how they are related has not always been clear and so, in this paper, we study the basis of them both and the possible relation between them.

### Emotional Intelligence (EI)

Emotion can be understood as an internal event that energizes human behavior so we can respond to our context by approaching or distancing, depending on whether emotions take a positive or negative hedonic tone (Ekman and Davidson, 1994). Intelligence has been explained as the ability to adapt to this context (Sternberg, 2013). By connecting them both, EI would be defined as people’s ability to perceive, understand, regulate and express such emotional events in an adaptive way (Celma-Pastor and Rodríguez-Ledo, 2017). Therefore, EI is a construct that explains how emotions are perceived, regulated and expressed with more or less skill. This understanding improves by the theoretical model proposed by the trait model of Petrides et al. (2007a), which states that EI is based on some personality traits that can consequently be assessed. Such a trait model of EI has been proposed (Petrides et al., 2007a), and it indicates that EI can also be known as an emotional self-efficacy trait, which refers to a constellation of emotion-related self-perceptions and dispositions located at lower levels of personality hierarchies (Petrides et al., 2007b). Moreover, the possibility of improving EI subcomponents has also been demonstrated when the design and implementation of programs follow appropriate quality measures (Durlak et al., 2011). EI has not always been the only objective to be promoted by SEL Programs as they sometimes aim to improve other related aspects, such as mindfulness, which is addressed specially in this study.

### Mindfulness

The educational psychology field is concerned about, and interested in, studying EI, and research findings support the notion that better motivation and engagement (Saeed and Zyngier, 2012), autonomy (Grolnick and Ryan, 1987) and attention abilities (Kruschke, 2003) enhance student learning. So, educators feel that these three aspects are important and should be promoted in their classrooms (Deci and Ryan, 2008). Consequently, in different countries, many educational models have faithfully reflected this socially desirable norms. They have traditionally rewarded young people who pay attention for long time periods, who are not distracted, and who know how to control their impulses to speak or move (Rodríguez-Ledo and Orejudo-Hernández, 2017). However, this model is changing, largely due to the emergence of more restless and impulsive students, and even to an increase in developmental disorders with a strong attentional component, such as Attention Deficit Disorder and Hyperactivity (ADHD). As indicated by Pagès and Reñé (2008), one of the possible causes could be the high stimulation that today’s students receive via many diverse channels, such as regular interaction with screens, especially the Internet. This overstimulation may be the consequence of tablets, computers, video games, especially mobile phone overuse, which are elements that continually require attention. Moreover, several other authors explain that it stems from the culture of immediate reinforcement and intolerance to frustration (García et al., 2011). Apart from looking for causes, it is believed that psychologists and educators should focus on interventions to improve attention and to reduce impulsivity in order to, therefore, revert this tendency. Following this goal, some interventions have attempted to improve such mindfulness competence in youngsters. For instance, Bluth and Eisenlohr-Moul (2017) have found that the implementation of the 8-week MindUp program achieves improvements in perceived stress, resilience, curiosity/exploration and gratitude, and that mindfulness covaries with perceived stress, depressive symptoms and anxiety. Other interventions in the same direction have proven that the implementation of such programs with young adolescent students is related to improvements in areas like: sustained attention (Zeidan et al., 2010), academic performance (León, 2008), reduced anxiety (Sugiura, 2004), reduced aggressiveness (Lantieri, 2009), decreased ADHD symptoms (Lantieri, 2009), lower burnout levels and enhanced motivation to the task or engagement (De La Fuente et al., 2010), and also better levels of emotional well-being (Hamilton et al., 2006). These are some of the most characteristic examples of improvements. An experienced reader can observe that many of the advantages of applying the aforementioned mindfulness techniques are related to improvements in components related to EI and their competences. In fact, other researchers have pointed out the importance of attention for developing the implicit cognitive capacities that make up EI (Mestre et al., 2009).

Following this goal, the best way to enhance attentional mechanisms has been researched in many different ways. Traditionally, the classic definition of attention by James (1890) has been taken as a valid one. He understood attention as the mind clearly and vividly taking several possible objects of thought that simultaneously appear. This attentional mechanism has been considered an essential element in the information management process as it influences the selection of information and establishes priority in processing (Laberge, 1995). In addition, a consensus exists about the three main functions of attention in human beings (Posner et al., 2007): the alert function, the orientation function and the executive function. The first refers to the state of tonic and phasic preparation to attend to new stimuli. The second refers to the selection of which of these stimuli are salient and is, therefore, adaptive to the process. The third function is related to something more complex: i.e., the supervisory system. This system is in charge of planning the whole process, of maintaining attention during this process, and evaluating performance by correcting it for improvement on future occasions (Chica Martínez and Checa Fernández, 2015). Apart from such functions and to look further at diverse attentional mechanisms and their adaptive sense in real practice, different authors defend dichotomous categories such as (Roselló, 1998): internal *versus* external, voluntary *versus* involuntary, open *versus* covert, divided *versus* selective, visual *versus* auditory, etc. Of these categories, a specific type of voluntary attention drew our attention: mindfulness. If voluntary attention is defined as that which can be directed according to our will, and to both the outside (sensations) and the inside (ideas and emotions) (León, 2008), mindfulness is defined as the ability to focus on the present moment in an active and reflective way (Vallejo, 2006).

In order to delimit the concept, several studies have emerged to define it as conscience without judgment, which develops moment after moment by a kind of non-reactive attention that is open and without prejudices in the present time (Kabat-Zinn, 2007), and which allows the awareness of our internal and external experiences without rejecting anything and without clinging to anything (García, 2007). Even more comprehensively, Grossman et al. (2004) define mindfulness as the ability to maintain moment-to-moment attention to emotional and social events, which may be our own or others, and in a non-evaluative fashion (Grossman et al., 2004). Bishop et al. (2004) consider that mindfulness is carved by two components: (1) the self-regulation of attention that is maintained in the immediate experience, which thus allows mental events to be recognized in the present moment; (2) particular orientation toward one’s experience in the present moment, characterized by curiosity, openness and acceptance. Therefore, according to Bishop et al. (2004), mindfulness skills are related to three areas of attention: sustained attention, selective attention, and the ability to direct and exchange the focus of attention from one idea to another. Shapiro et al. (2006) attempt to identify the mechanisms of action that are the basis of mindfulness by proposing two research lines: (1) studies that seek to separate and compare the various active ingredients in mindfulness interventions and their relation to other constructs, such as cognitive or emotional capacities; (2) studies that analyze the mindfulness construct itself to determine whether mindfulness development is what really leads to the positive changes that have been observed in many fields. The present study seeks both objectives, while it also attempts to base the relation between basic emotional competences and those of full attention, and to study whether it makes sense working such competences together in the classroom to obtain satisfactory results.

### EI and Mindfulness

When arguing about what EI can be like and, in some way, in relation to this ability to maintain moment-to-moment attention in a non-evaluative manner, Brown and Ryan (2003) indicate that the clarity of perception of own emotional states improves through training in mindfulness techniques. Besides, it is known that a direct relationship exists between training with mindfulness techniques and attention, clarity and emotional repair dimensions (De La Fuente et al., 2010), as well as emotional regulation (Ramos et al., 2009). Along the same lines, Davidson has conducted studies with expert Buddhist monks in mindfulness by demonstrating the primordial importance that mindfulness has when it comes to modifying emotional states voluntarily, and has directly correlated this attentional capacity and emotional regulation (Davidson, 2012). Davidson has also demonstrated how training in mindfulness helps develop more positive and adaptive emotional profiles (Goleman and Davidson, 2017). According to Schoeberlein (2012), the implementation of mindfulness techniques with teachers shows improvements in receptivity to student requirements, promotes emotional balance, contributes to stress management, favors personal relationships, improves the classroom climate and contributes to general well-being. The same author has also found that when students work with mindfulness, it favors their willingness to learn, fosters academic performance, reinforces attention and concentration, reduces anxiety about exams, improves participation in the classroom, promotes impulse control, provides tools to help reduce stress, improves emotional learning, promotes prosocial behavior and supports holistic wellness (Schoeberlein, 2012). Moreover, mixed EI and mindfulness intervention programs, such as that implemented by (Schonert-Reichl et al., 2015), typically find the improvements described in their paper, namely: greater empathy, better emotional control, increased peer acceptance, higher mindfulness competence and even better cognitive and stress control, among others (Schonert-Reichl et al., 2015).

The underlying process that explains how mindfulness achieves such advantages for EI and to enhance some of its components is still being debated. Ramos et al. (2009) explain that training in mindfulness involves exposure to negative emotions that favor habituation to them. Vallejo (2006) indicates that people can change tendencies of automatic response to certain emotional experiences thanks to training in mindfulness, which allows people to respond with other new and more positive behavioral repertoires as a result of quiet reflection. As supported by Holen and Halvor (2007), perhaps the continuous practice of such full consciousness would prepare us against adversity, while reducing tension, fears and worries through the progressive disengagement of thoughts, sensations and emotions. This would thus be a very useful and effective emotional self-regulation mechanism. Shapiro et al. (2006) indicate that mindfulness techniques make users more adaptable, flexible and suitable for the context by developing the re-perceiving mechanism to facilitate reactivity patterns. This is achieved by the fact that the impact allows us to see the current situation as it is at the present time, which helps us to act accordingly by avoiding the thoughts, emotions and maladaptive behavior that we were used to before developing our full consciousness (Shapiro et al., 2006).

### Enhancing Mindfulness Through the SEA Program

After considering the potential of implementing mindfulness in the classroom, the authors of this study decided to design a program to enhance mindfulness, along with some competences based on participants’ EI. The SEA Program to develop emotional and mindfulness competences was designed (Celma-Pastor and Rodríguez-Ledo, 2017) in an attempt to fulfill the best conditions (Durlak et al., 2011) and to promote the development of EI, understood as the capacity to perceive, understand, regulate and express one’s own and others’ emotional events adaptively (Celma-Pastor and Rodríguez-Ledo, 2017). It also aimed to enhance mindfulness, or the ability to maintain moment-to-moment attention on emotional and social events, either our own or those of others, and in a non-evaluative manner (Grossman et al., 2004). Such a program is meant to be implemented for 1 h/week throughout an academic year (9 months) by following the 18 sessions that compose it. These sessions have been designed to develop such EI and mindfulness by reflecting on participants’ current level and by actively working to improve subcomponents according to the described constructs. Mindfulness capacity is specifically developed by following 10 short body scanning and meditation sessions to address external or internal events, as well as other activities, to enhance youngster’s awareness or to maintain attention to specific inputs.

According to this twofold objective, which is exploratory before and becomes experimental later, we studied the relation between the relationship between EI and mindfulness in a first phase to discover whether both were related in our sample, and if it made sense to implement them both at the same time. Afterward, the program was implemented in a second phase and any improvement in the mindfulness competence of the participants in the present study was analyzed experimentally by comparing pre/post-test measures. In this way, the first hypothesis that guided our research in the first phase was: mindfulness and EI are closely related constructs. The second hypothesis, which was characteristic of the second phase and related to the improved ability of mindfulness, was proposed: applying the SEA Program for one academic year will improve the mindfulness of the participants in this implementation.

## Materials and Methods

### Participants

Our study sample consisted in 156 adolescents aged 11–14 years, distributed into the six class groups of the first grade of Compulsory Secondary Education (ESO in the Spanish education system) at a public high school in a working-class neighborhood in the city of Zaragoza (Spain). Four of these class groups were randomly assigned to the experimental condition (108 participants) and the others to the control (48 students). In gender terms, 86 were male (55.1%) and 70 were female (44.9%). Of the 108 experimental participants, 58 were male (53.7%) and 50 were female (46.3%). Of the 48 control participants, 28 were male (58.3%) and 20 female (41.7%). The gender distribution for the two conditions was statistically and significantly equivalent (*X*^2^ = 0.592 and *p* > 0.05). From the original number of participants (156), the study lost four of the participants, being 152 the final number of participants in the post-test. The sample’s socio-economic and cultural status was medium. The parents, mothers and legal guardians of the participants were informed about the research characteristics in writing and during a meeting. They all received explicit consent to carry out this study. The anonymity of the answers and scores of all the participants were guaranteed and respected.

### Instruments

The instruments used to evaluate the relationship between EI and mindfulness, and the effects of intervention on mindfulness, were chosen from among the many possible options by considering the study sample’s age, the model that lies behind such instruments and, of course, the validity of tests. Since our study participants were Spanish youngsters aged 11–14 years old, and according to the model of EI behind the SEA program, the selected instruments were the following:

The emotional development questionnaire for secondary CDE-SEC (Álvarez et al., 2001). This self-report questionnaire consists of 35 items that must be answered according to the degree of agreement or disagreement, 0 meaning completely disagree and 10 completely agree. The EI dimensions assessed by this questionnaire were: (1) Emotional awareness: Ability to become aware of one’s own emotions, including the ability to understand the emotional climate of a given context; (2) Emotional regulation: Ability to use emotions appropriately and have good “coping” strategies, ability to self-generate positive emotions; (3) Emotional autonomy: characteristics related to emotional self-management, such as self-esteem, positive attitude in life, responsibility, ability to critically analyze social norms, ability to seek help and resources, and personal self-efficacy; (4) Social competences: ability to maintain good relationships with other people and to master basic social skills, effective communication, respect, pro-social attitudes, assertiveness, etc.; (5) Competences for life and well-being: Ability to adopt appropriate and responsible behavior to solve personal, family, professional and social problems, oriented toward improving the well-being of personal and social life. A total emotional competence score can also be obtained, which results from the average of all the assessed variables. Test reliability varied between α = 0.79 and α = 0.82 for each dimension (Escoda, 2016) and it has been designed and tested for a sample of Spanish youths.

The emotional intelligence questionnaire, youth version EQi-YV (Ferrándiz et al., 2012). The EQi-YV is a self-report questionnaire designed to measure the EI of children and adolescents aged from 7 to 18 years. It is based on the original EQ test (Bar-On, 1997) and has been adapted to Spanish samples. It is a Likert scale whose 60 items give rise to five large EI dimensions that allow subjects’ following emotional and social characteristics to be studied: (1) Intrapersonal ability: ability to understand their own emotions and their communication with others; (2) Interpersonal ability: ability to understand and appreciate others’ emotions; (3) Managing emotions: ability to direct and control one’s emotions; (4) Adaptability: flexibility and effectiveness to adapt to the social environment and to solve conflicts; (5) General mood: ability to take a positive attitude toward life. It is a wide inventory that provides information about emotional and social competences, and it allows to draw a total social and affective profile known as the total emotional quotient. The validation in the Spanish sample obtains a reliability that oscillates between α = 0.63 (intrapersonal competence) and α = 0.80 (mood) for all five dimensions (Ferrándiz et al., 2012).

The BAS3 socialization battery (Silva and Martorell, 2001). BAS3 consists of 75 items in this self-application version designed for Spanish samples. It evaluates five dimensions that allow us to obtain participants’ social behavior profile and their social adjustment, namely: (1) Consideration with others: it detects social sensitivity or preoccupation of others, in particular of those with problems and who are rejected or postponed; (2) Self-control in social relations: it includes a clearly bipolar dimension that represents, on its positive pole, compliance with social rules and norms that facilitates coexistence in mutual respect, and on its negative pole, aggressive and stubborn behavior and indiscipline; (3) Social withdrawal: it detects both passive and active withdrawal from others, until it arrives at a clear isolation (external); (4) Social Anxiety/Shyness: different manifestations of anxiety are detected together with reactions of shyness in social relationships; (5) Leadership: ascendancy, popularity, initiative, self-confidence and service spirit are detected. It is a highly reliable test that presents an average internal consistency of α = 0.75 and a test–retest stability of α = 0.57 measured with Cronbach’s alpha (Silva and Martorell, 2001).

The empathy scale for children and adolescents (ESCA) (Bryant, 1982; Mestre et al., 1999). This scale consists of 22 items of yes–no responses that evaluates general cognitive and affective empathy according to participants’ agreement or disagreement in 22 situations. It is an adaptation of the infantile and adolescent population (from 11 years of age) of the scale for adults by Mehrabian and Epstein (1972) carried out by Bryant (1982). Specifically, it is a scale that provides a general empathy index, whose internal consistency of the original scale is 0.67 (Bryant, 1982). With the Spanish sample, the scale presents a test–retest reliability between α = 0.75 and α = 0.77, depending on the sample used by the Spanish research from which it was obtained (Mestre et al., 1999).

The mindfulness scale for school scope (León, 2008). This self-report scale is designed especially for young high school students aged 11–15 years. It must be answered in a Likert format with five intervals from 1 to 5, which represent a continuum that goes from “never” to “always” by evaluating three mindfulness dimensions: (a) kinesthetic mindfulness, which refers to the ability to notice movement and motor actions; (b) external mindfulness, which refers to the ability to direct attention to external elements, attention for observation; (c) interior mindfulness, which refers to the ability to direct attention to the intellectual, to the world of ideas, emotions and feelings, which would be attention for introspection. It also offers a total score in the mindfulness product of the sum of the previous three variables. This questionnaire’s general internal consistency is α = 0.84. For each component, the author reports a consistency of α = 0.74 for the kinesthetic mindfulness dimension, α = 0.60 for the external mindfulness dimension and α = 0.66 for the interior mindfulness dimension. Test–retest reliability is *r* = 0.78 at 4 weeks. The exploratory factorial analysis reports that the three factors explain almost 53% of total variance as follows: 35.20% for kinesthetic mindfulness, 9.50% for external mindfulness and 8.10% for internal mindfulness.

### Procedure

First of all, the relationship between EI and mindfulness was studied to look further at how both are related, and to thus determine the suitability of working with them together in implementing the program. In a second phase, a quasi-experimental pre-/post-test design was used with a control group to evaluate the effectiveness of the intervention program for participants’ mindfulness. Those who applied the program (applicators) in the four class groups that make up the experimental group were the tutors of these class groups, who implemented the program sessions. Sessions were always held at the same time in the morning as part of the tutorial subject (the Spanish education system includes 1 h/week designed to work many different non-academic aspects). The times to apply the experimental groups and controls were balanced so that the application hours were distributed equally during teaching hours. It took 9 months to complete this educational intervention with the participants, during which 55-min sessions were held when the SEA Program was organized. Concretely, the 18 sessions of the SEA program were implemented during October 2014 and June 2015. The diagram of the flow of the study shows in detail dates of the educative intervention accomplished (see Table 1).

**Table 1:** Diagram of the flow of the study.

Period	Implementation
14th October 2014 – 13th November 2014	Tutors training and pretest assessment
17th November 2014 – 22nd May 2015	SEA Program implementation
25th May 2015 – 12th June 2015	Post-test assessment

During 10 of the SEA Program sessions, mindfulness was implemented in the first 10–15 min of the session. The mindfulness techniques, which formed part of the program, were designed to be useful in the participants’ real world by seeking their real practical use and attempting to transfer them to their real lives. These mindfulness techniques used meditation techniques to improve internal and external attention. Specific techniques were also used to improve the studied mindfulness subcomponents by seeking improvements in sustained attention through frets and matrices, in the association of students’ life spaces to a higher level of attention and a lower level of activation, and by reducing students’ activation tone prior to lessons (see Celma-Pastor and Rodríguez-Ledo, 2017). Half of these sessions involved mindfulness meditation. Thus, the ability to maintain attention openly, both inside and outside, according to what the CD instructions expected, used to guide this activity, was enhanced by anchoring to different aspects: breathing, association of words, body scanner, etc.

Implementing the program’s activities was preceded by training given of the tutors who would hold the sessions. This training was carried out by external experts in the area and dealt with the importance of enhancing EI and mindfulness in the classroom, the program’s basic theoretical model and the specific application techniques for the different sessions and activities. A weekly follow-up was also carried out during formal and informal meetings, held with experts and applicators to verify if the program was being correctly implemented, to offer help and advice based on specific activities.

The instruments used to collect the data from both phases were administered by the tutors of the class groups under all the experimental conditions. All the instruments were applied twice, one pretest before implementation commenced and a post-test once all the program sessions had finished. Written informed consent to collect data was obtained from the parents/legal guardians of all the participants. The data analyzed herein were those collected by the pretest measurement.

### Data Analysis

Given the normal distribution of errors and the linear relationship of the dependent and independent variables, the reported results about EI and mindfulness competences were examined by a general linear model analysis. To study the relationship between the EI and the mindfulness scores in the first phase, the analysis used bivariate correlations by employing Pearson’s r-statistics, where the correlations between 0 and 0.3 were low, those between 0.3 and 0.6 were the medium, and those between 0.6 and 0.9 were high (Field, 2013). In all cases, the pretest measures of the different tests were used so that the implementation effects could be controlled.

In the second phase, the ANCOVA univariate model was applied per measure, where the dependent variable was the post-test measure, the covariate was the pretest measure and the fixed factor was the experimental condition. The statistics used to measure the effect were Pillai’s trace (*F*) and $\eta_{p}^{2}$, which measure the effect size, where $\eta_{p}^{2}$ < 0.06 was small, $\eta_{p}^{2}$ > from 0.06 to <0.14 was medium and $\eta_{p}^{2}$ > 0.14 was high (Field, 2013).

## Results

In order to verify or refute the research hypotheses, the obtained results were analyzed, first in relation to the link between the EI and mindfulness competences, which were characteristic of the study phase 1, and to then look further at the improvements found after applying the program in phase 2.

### Phase 1: The Relation Between EI and Mindfulness

Table 2 shows the bivariate correlation scores, measured with Pearson’s r, between the EI variables and the mindfulness variables. All the sample participants (both the control and experimental ones) were analyzed, where *N* = 152.

**Table 2:** Bivariate correlation scores.

	External	Internal	Kinesthetic	Total of
	mindfulness	mindfulness	mindfulness	mindfulness
**Emotional Intelligence (CDE-SEC)**				
Emotional awareness	–0.073	0.305**	0.121	0.129
Emotional regulation	–0.015	0.111	0.185*	0.116
Emotional autonomy	0.041	0.165*	0.142	0.138
Social competences	–0.023	0.126	0.024	0.045
Competences for life and well-being	0.189*	0.310**	0.107	0.233**
Total score of EI	0.040	0.278**	0.168*	0.188*
**Emotional Intelligence (EQi-YV)**				
Intrapersonal ability	0.015	0.240**	0.098	0.134
Interpersonal ability	0.138	0.144	0.079	0.144
Managing emotions	0.116	0.054	0.175	0.148
Adaptability	0.090	0.029	0.136	0.110
General mood	0.100	0.221*	0.196*	0.208*
Total emotional quotient	0.151	0.198*	0.220*	0.232*
**Empathy (ESCA)**				
General empathy	0.205*	0.231**	0.004	0.165*
**Social adjustment and abilities (BAS3)**				
Consideration with others	0.096	0.207*	–0.035	0.095
Self-control in social relations	0.224**	0.145	0.202*	0.235**
Social withdrawal	0.141	–0.157	–0.025	–0.006
Social anxiety/shyness	0.215**	–0.054	0.005	0.074
Leadership	0.096	0.118	0.019	0.089

*^∗^The correlation is significant at the 0.05 level (two tails). ^∗∗^The correlation is significant at the 0.01 level (two tails).*

By analyzing the statistically significant correlations according to the different study dimensions, we noted that for the EI variables, the external mindfulness dimension positively correlated only with the variable competences for life and well-being (*r*_152_ = 0.189 and *p* < 0.05). The internal mindfulness variable correlated positively with a variety of EI-related variables: emotional awareness (*r*_152_ = 0.305 and *p* < 0.01), emotional autonomy (*r*_152_ = 0.165 and *p* < 0.05), competences for life and well-being (*r*_152_ = 0.310 and *p* < 0.01), intrapersonal ability (*r*_152_ = 0.240 and *p* < 0.01) and general mood (*r*_152_ = 0.221 and *p* < 0.01). This correlated with both the total EI score (*r*_152_ = 0.278 and *p* < 0.01) and the total emotional quotient (*r*_152_ = 0.198 and *p* < 0.01). Finally, kinesthetic mindfulness correlated positively with the variables of emotional regulation (*r*_152_ = 0.185 and *p* < 0.05) and general mood (*r*_152_ = 0.196 and *p* < 0.05). This also correlated with both the total emotional competence score (*r*_152_ = 0.168 and *p* < 0.05) and the total emotional quotient (*r*_152_ = 0.220 and *p* < 0.05). We also noted that the total mindfulness score correlated positively with the EI variables competences for life and well-being (*r*_152_ = 0.233 and *p* < 0.01), interpersonal ability (*r*_152_ = 0.217 and *p* < 0.05) and general mood (*r*_152_ = 0.208 and *p* < 0.05). It also correlated with both the total EI scores: total emotional competence (*r*_152_ = 0.188 and *p* < 0.05) and the total emotional quotient (*r*_152_ = 0.232 and *p* < 0.05).

We also observed a positive and statistically significant correlation between the variable empathy and the external (*r*_152_ = 0.205 and *p* < 0.05) and internal (*r*_152_ = 0.231 and *p* < 0.01) mindfulness dimensions, and with the total mindfulness score (*r*_152_ = 0.165 and *p* < 0.05).

Finally, after analyzing the statistically significant correlations for the variables of social abilities and social adjustment, we saw that the external mindfulness dimension correlated positively with the self-control variables in social relations (*r*_152_ = 0.224 and *p* < 0.01) and with social anxiety/shyness (*r*_152_ = 0.215 and *p* < 0.01). The internal mindfulness variable correlated positively with the variable consideration with others (*r*_152_ = 0.207 and *p* < 0.05). The kinesthetic mindfulness dimension correlated positively with the variable self-control in social relations (*r*_152_ = 0.202 and *p* < 0.05), whereas the variable total mindfulness correlated only with the socialization dimension assessed in the variable self-control in social relations (*r*_152_ = 0.235 and *p* < 0.01).

### Phase 2: Effects of the Implementation on Mindfulness

In order to support or refute the second hypothesis, the changes found in the mindfulness competence after implementing the SEA Program were analyzed. Table 3 shows the descriptive statistics scores of the different factors of the variable mindfulness measured herein with its pretest and post-test measures for each experimental condition.

**Table 3:** Descriptive statistics scores.

	Pretest		Post-test	
	*Mean*	*SD*	*Mean*	*SD*
**Experimental condition**				
Kinesthetic mindfulness	13.76	3.955	13.81	4.426
External mindfulness	15.31	3.670	14.46	3.601
Internal mindfulness	15.76	3.257	16.50	2.686
Total of mindfulness	44.84	8.766	44.07	9.841
**Control condition**				
Kinesthetic mindfulness	12.81	5.037	11.93	3.963
External mindfulness	13.94	4.508	13.78	4.005
Internal mindfulness	16.30	3.230	15.74	3.593
Total of mindfulness	43.04	11.055	41.22	9.867

*Experimental group *N* = 108 and control group *N* = 48.*

After presenting the descriptive data, the results obtained after applying the ANCOVA univariate general linear model analysis are shown in Table 4. In it, the pre/post-test changes, presumably through educational intervention, introduced into the mindfulness competences of the participants were observed. They were measured by Pillai’s trace (*F*), and always with 1 degree of freedom, and their effect size ($\eta_{p}^{2}$) and their statistical significance (*p*). Note *N* = 108 for the participants of the experimental group and *N* = 48 for the controls.

**Table 4:** General linear model scores (ANCOVA).

	*F*	$\eta_{p}^{2}$	*p*
Kinesthetic mindfulness	4.326^∗^	0.029^∗^	0.039
External mindfulness	0.000	0.000	0.987
Internal mindfulness	4.979^∗^	0.033^∗^	0.027
Total of mindfulness	1.813	0.012	0.180

*^∗^The effect is significant with a level of significance of <0.05.*

As noted, the results offered significant differences in two of the three measured mindfulness variables. Specifically, effects were observed in the experimental group on those in the control group for kinesthetic mindfulness (*F*_156_ = 4.326, $\eta_{p}^{2}$ = 0.029 and *p* < 0.05) and internal mindfulness (*F*_156_ = 4.979, $\eta_{p}^{2}$ = 0.033 and *p* < 0.05).

Regarding gender differences, statistical differences were observed between the male and female participants in the pretest of some variables: empathy and social shyness, where the female participants obtained better marks; kinesthetic mindfulness, where the male participants obtained higher scores. However, the data analysis did not find any interaction between gender and the experimental condition. So, we concluded that such gender differences are distributed equally in both the control and experimental conditions. Nor were any statistically significant differences found in improvement between males or females according to the pre/post-tests of their mindfulness capacity as the male and female participants showed similar improvements after the program.

## Discussion

The objective of the present paper was twofold: an exploratory study in a first phase of the relation between the EI construct and the mindfulness construct; in a second phase, to assess the impact on the participants’ mindfulness competences of implementing the Emotional Competency Development SEA Program.

Regarding the first objective, typical of phase 1, we detected that EI and mindfulness are related in many of the variables measured by the instruments. In particular, we suggested that having a good mindfulness competence is related to having a good general EI level and a higher total emotional quotient. This finding is consistent with those studies that have found a relationship between maturing attentional abilities in children and a greater capacity to manage one’s emotional states (Mestre et al., 2009; Davidson, 2012; Eisenberg, 2013).

A relation of this type of full attention or mindfulness and EI was found to be more profound according to the results provided herein. According to them, the participants with better capacities to fully pay attention to external events are those with better skills for life and well-being. They are also more empathetic and are better regulated socially, although they may be too shy sometimes. Those participants with better competences to fully pay attention to internal events are more aware of their emotions, are more emotionally autonomous, and are more competent to deal with personal and social problematic situations with better intrapersonal skills and a better overall mood. They are also more empathetic and consider the opinions and wishes of others more, which are important social aspects in any educational environment. In addition, those who better attend to personal and internal events obtain better overall EI scores and can benefit from the traits related to such intelligence.

Finally, and having taken into account the general ability to attend with mindfulness of an external origin, of an internal cognitive origin, or one that is even body-kinesthetic related, a relation with EI was shown. The young participants in our sample who enjoy having more mindfulness competences are people with better skills for life and well-being, and they are more empathetic and better regulated socially, which make them youngsters with better total EI and a higher emotional quotient. This demonstrates a relation between mindfulness and EI, which leads us to wonder whether enhancing both together in the classroom can, in fact, be more positive and effective.

According to this second objective of phase 2 (i.e., implementing the SEA Program with its 18 sessions to enhance some component based on EI and mindfulness), we obtained interesting results. The pre–post evaluation made with the Mindfulness Scale for the School Scope (León, 2008) informed us about the significant effect on the experimental participants compared with the controls for the ability to mindfully attend the interior or the ability to pay attention to the intellectual, to the world of ideas, emotions and feelings. This effect also came over in the capacity of participants’ kinesthetic attention or their capacity to make movement and motor actions. Rather than acting as an enhancer, this effect was protective because, according to the obtained data, the ability to fully attend seemed to deteriorate which, in turn, deteriorated this competence. The implemented program was predictably the main aspect to work as a protective factor by absorbing or cushioning this deterioration. The deterioration detected in teenagers’ ability to fully attend was consistent with the tendency to worsen, as consistently observed and reported by teachers and educators informally since they started year 1 of secondary education to even higher grades, such as high school year 3 (Greenberg et al., 2003). If aspects such as self-esteem have been found to decrease in adolescence, especially in early stages (Sanchez, 2009), and as EI also seems to do so (Greenberg et al., 2003), attentional aspects may deteriorate, which may be recovered throughout the adolescent stage. This has been confirmed by several studies which have found precisely this attentional worsening in adolescents compared to children and adults (Santos Cela, 2013). If general attention worsens, probably the ability to mindfulness also does, so educational interventions to curb this worsening could act as protective factors from this decline. Thus, the SEA Program proved successful in achieving this in the assessed sample.

The results obtained about the effect of the program support the program itself, as a properly designed one that obtains results which encourage educators to continue implementing it in the classroom so they can continue strengthening attention abilities or can stop typical deterioration in teenager stages. It has also been discovered that a program which works the EI subcomponents together with brief mindfulness techniques can obtain results that improve the latter. Findings by different researchers confirm that by improving mindfulness in the classroom, students’ EI (Brown and Ryan, 2003; Ramos et al., 2009; Arias, 2010) and their mindfulness ability (De La Fuente et al., 2010) also improve.

It should be noted that this study contains certain limitations which future researchers are encouraged to overcome. One is related to our sample size and the studied age range. Larger samples may provide more conclusive data. Likewise, we encourage discovering possible improvements in implementing such aspects with other sample types with different ages or idiosyncratic characteristics, such as young people with ADHD. In addition to new and expanded samples, we also encourage researchers to implement the 10 SEA Program sessions that work mindfulness separately by evaluating if the obtained results can be replicated. Although conclusive results were found, it is the students themselves who report their level of competence by using self-report tests. Future researchers are encouraged to use objective measures to evaluate participants’ level of mindfulness. As no such objective test was used with the assessed sample, the authors of this study chose the test as it was thought to be best for our sample evaluation, which was that used herein. It is noteworthy that although the found results could appear encouraging considering the brief intervention made, the improvements obtained a small effect size and low correlations, which is something to be considered by future applicators. Perhaps longer and more cross-sectional implementations may obtain higher scores. We therefore encourage interventions in developing EI-mindfulness during longer interventions that are longitudinal to check whether this protector effect remains over time.

## Conclusion

Beyond possible future improvements in research terms, the main objective of all experts in Education and Developmental Psychology is to improve the education system of their respective countries as it is capital to develop people with a good set of competences for life, including mindfulness. It is known that young people with better competence to mindfully attend will be predictably more attentive, less anxious, will better control their impulses (Sugiura, 2004; Zeidan et al., 2010; Schoeberlein, 2012) and display better academic performance (León, 2008), with lower burnout levels and will feel more motivated by the task (De La Fuente et al., 2010), more prosocial and less aggressive (Lantieri, 2009). They will also have better emotional well-being levels (Hamilton et al., 2006; Schoeberlein, 2012). It has been suggested that such a mindfulness competence can be enhanced by direct intervention in the classroom, which is relatively short and seeks to promote emotional aspects, together with the ability to fully attend time after time. It has been shown how EI correlates with mindfulness and, thus, by working both, we will perhaps further enhance them both. Following Shakir et al. (2017), we also thought about extending training in mindfulness and/or EI techniques to other fields where professionals can easily benefit from it, such as medical professionals and in health care environments. However, we must set a short-term to mid-term goal to continue improving future adults’ competences. Therefore, and by considering that if our obligation as psychologists and educators is to train for personal development, the SEA Program can be a successful option to achieve this goal as it becomes an intervention proposal to join many others who seek the long-awaited goal that every educational system yearns for: developing competent people for all the scopes of their lives.

## Ethics Approval

The authors hereby state that they have not received any payment for this submitted work and have no financial relationship with any organization that could be perceived to influence or give the appearance of potentially influencing what we have written in the submitted work.

The SEA intervention program has been published in TEA Editorial (see Celma-Pastor and Rodríguez-Ledo, 2017) for the interest of possible applicators, but no data have been published in it. Besides this, we have no other relationships or activities that readers could perceive to have influenced or to give the appearance of potentially influencing we have written in the submitted work.

No ethics approval was required for this research since the Spanish public education system and national regulations require no such approval.

## Author Contributions

All authors listed have made a substantial, direct and intellectual contribution to the work, and approved it for publication.

## Conflict of Interest Statement

The authors declare that the research was conducted in the absence of any commercial or financial relationships that could be construed as a potential conflict of interest.
